# Enhancing Our Understanding of Plant Cell-to-Cell Interactions Using Single-Cell Omics

**DOI:** 10.3389/fpls.2021.696811

**Published:** 2021-08-05

**Authors:** Sandra Thibivilliers, Marc Libault

**Affiliations:** Department of Agronomy and Horticulture, Center for Plant Science Innovation, University of Nebraska-Lincoln, Lincoln, NE, United States

**Keywords:** transcriptomics, single-cell omics, multi-omics analyses, spatial transcriptomics, cell-to-cell interactions

## Abstract

Plants are composed of cells that physically interact and constantly adapt to their environment. To reveal the contribution of each plant cells to the biology of the entire organism, their molecular, morphological, and physiological attributes must be quantified and analyzed in the context of the morphology of the plant organs. The emergence of single-cell/nucleus omics technologies now allows plant biologists to access different modalities of individual cells including their epigenome and transcriptome to reveal the unique molecular properties of each cell composing the plant and their dynamic regulation during cell differentiation and in response to their environment. In this manuscript, we provide a perspective regarding the challenges and strategies to collect plant single-cell biological datasets and their analysis in the context of cellular interactions. As an example, we provide an analysis of the transcriptional regulation of the Arabidopsis genes controlling the differentiation of the root hair cells at the single-cell level. We also discuss the perspective of the use of spatial profiling to complement existing plant single-cell omics.

## Introduction: How Single-Cell Approach Can Help to Enhance Our Understanding of Plants as Biological Systems?

**Figure 1:**
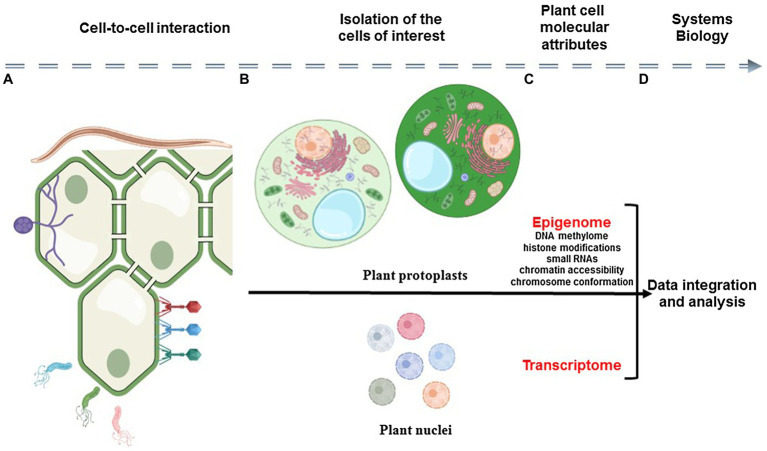
Schematic representation of the analysis of plant cell-to-cell interaction using single-cell omics technologies. Plant organs are composed by physically interacting somatic cells that constantly adapt to their biological environment **(A)**. The analysis of the epigenomic and transcriptomic profiles of isolated plant cells (i.e., epigenome and transcriptome) requires the isolation of individual cells and nuclei **(B)** before the construction of DNA sequencing libraries **(C)**. Therefore, the relative position of the cell in the organ and its interaction with somatic, microbial, and pathogenic cells is lost. A systems view of plant cellular communication would require the capture of cellular modalities in the context of the morphology of the plant sample before their integration using computational tools **(D)**.

Plants are complex and very dynamic biological systems composed of various cell types that communicate together and constantly respond to their environment. Therefore, to better understand plants as biological systems, there is a need to understand the contribution of each cell to the biology of the organism, to reveal the unique and dynamic response of each plant cell to environmental stimuli, and to characterize how cell-to-cell communication plays a role in controlling these responses. To reach such knowledge, plant scientists must gain molecular information from each cell composing the tissue/organ/plant and analyze this information in the context of the spatial organization of the organ and interaction of the cells (Figure 1).

The emergence of single-cell omics technologies and their recent application on plant organs now enable the characterization of the molecular attributes of thousands of cells in one single experiment (Rich-Griffin et al., 2020; Shaw et al., 2021). For instance, recent studies reported the establishment of Arabidopsis roots, stomatal cells, and maize anther cell transcriptomes at the single-cell level using isolated protoplasts or nuclei as input (Denyer et al., 2019; Jean-Baptiste et al., 2019; Nelms and Walbot, 2019; Ryu et al., 2019; Shulse et al., 2019; Zhang et al., 2019; Liu et al., 2020b; Farmer et al., 2021). To complement this first set of information, single-cell ATAC-seq was also applied on nuclei isolated from Arabidopsis roots and various maize organs to reveal the differential chromatin accessibility between plant cell types (Dorrity et al., 2020; Marand et al., 2020; Farmer et al., 2021). Recently, Farmer et al. (2021) integrated single-nuclei RNA-seq and ATAC-seq datasets to reveal the impact of chromatin accessibility in controlling gene expression and the differential organization of the Arabidopsis genome between cell types. While informative, these analyses require the abolition of the interactions between plant cells, a pre-requirement to the study of the molecular attributes of each plant cell. In this manuscript, we provide an update about the current limitations and advantages associated with the use of single-cell omics technologies to study plant cell biology notably in the context of cell-to-cell communication. As an example, we provide a comprehensive analysis of the differential transcriptional regulation between H and N cells, epidermal root cells differentiating into trichoblasts and atrichoblasts, respectively.

## Accessing Isolated Plant Biological Entities to Conduct Omics Analyses

Plant biologists face unique challenges when characterizing the molecular attributes of each plant cell. First, the presence of the cell wall prevents the isolation of plant cells. To overcome this first difficulty, several groups used enzymatic cocktails to digest the plant cell wall and release plant protoplasts. These protoplasts were used as input into microfluidic systems to generate single-cell barcoded cDNA libraries and establish the transcriptomes of the Arabidopsis root cells (Denyer et al., 2019; Jean-Baptiste et al., 2019; Ryu et al., 2019; Shulse et al., 2019; Turco et al., 2019; Zhang et al., 2019; Shahan et al., 2020; Song et al., 2020; Wendrich et al., 2020), stomatal cells (Liu et al., 2020b; Lopez-Anido et al., 2020), leaf phloem cells (Kim et al., 2020), and sperm cells (Misra et al., 2019), and the transcriptomes of the cells composing the rice root tip (Liu et al., 2021) and the maize shoot apical meristem (Satterlee et al., 2020), developing ears (Xu et al., 2021) and anther (Nelms and Walbot, 2019). However, the release of a representative and viable population of protoplasts would require constant optimization by taking into consideration the unique biochemical composition of the cell wall without compromising protoplast viability. Therefore, the establishment of a protoplast-based single-cell transcriptome might be restricted to a limited number of plant species, organs, and cell types. For instance, Shulse et al. (2019) noticed that the transcriptome of differentiated endodermal cells that are characterized by the suberization of their cell wall was missing in Arabidopsis root protoplast single-cell transcriptomic datasets (Shulse et al., 2019). It is also important to acknowledge that plant protoplasts are prone to bursting and that protoplastization leads to the activation of the expression of protoplast-induced genes (Birnbaum et al., 2003).

As an alternative to the use of plant protoplasts, plant nuclei were recently utilized to establish biologically meaningful transcriptomic information from the Arabidopsis root (Farmer et al., 2021; Long et al., 2021), inflorescences (Sunaga-Franze et al., 2020), and seeds (Picard et al., 2020), and the tomato shoot apex (Tian et al., 2020). Besides, the use of isolated nuclei revealed the transcriptome of Arabidopsis roots cells that were not captured by protoplast-based single-cell transcriptomes likely due to the limited digestibility of their cell wall (Farmer et al., 2021). Therefore, this method represents an alternative to the use of plant protoplasts to broadly access the transcriptome of plant cells from different species and organs (Sunaga-Franze et al., 2020; Thibivilliers et al., 2020; Thibivilliers and Libault, 2021). However, accessing a more limited pool of polyadenylated transcripts from an isolated nucleus compared to an entire cell would necessarily lead to the detection of a lower number of expressed genes per nucleus vs. per protoplast. Considering that isolated nuclei are also prone to RNA leakage when not properly manipulated, the depth of the nuclear transcriptome might be low when using damaged nuclei. On the other hand, the nuclear transcriptome could be considered as a snapshot of the dynamic transcriptional activity of the genes while the cellular transcriptome may represent an integration of gene activity over time [i.e., the half-life of the cellular mRNA is estimated at 9 h in human cells (Schwanhausser et al., 2011)]. Hence, the nuclear transcriptome should allow the characterization of the early and more subtle responses of the cell in response to stress. Nevertheless, despite its challenges, it has been demonstrated that the nuclear transcriptome is sufficient to decode the tissue heterogeneity to a similar level to the cellular transcriptome.

## Multi-Omics Approaches to Reveal Plant Cell Dynamics

Accessing one molecular modality of plant cells, such as their transcriptome, is a major milestone. However, to capture the entire diversity and subtle differences existing between cells and to reveal cell-type-specific regulatory networks and biological processes, there is a need to characterize and integrate different modalities at the single-cell level (Iacono et al., 2019; Hu et al., 2020; Jackson et al., 2020). Previous studies revealed changes in the patterns of histone modifications and gene expression of the Arabidopsis stomatal cells (Lee et al., 2019), and in the profile of methylation of the Arabidopsis root cell types (Kawakatsu et al., 2016). More recently, Dorrity et al. (2020) and Farmer et al. (2021) characterized the profiles of chromatin accessibility of Arabidopsis root cells at the single-cell level using microfluidic technology on isolated plant nuclei (Dorrity et al., 2020; Farmer et al., 2021). A similar study was also conducted on maize axillary buds, inflorescences, whole seedling, embryonic root tips, and post-embryonic crown roots cells (Marand et al., 2020). As a first effort in integrating various molecular markers of plant cells, Horvath et al. (2019) revealed that CG-methylated Arabidopsis genes are constitutively expressed (Horvath et al., 2019). More recently, Farmer et al. (2021) revealed the impact of the profiles of chromatin accessibility in regulating the transcriptional activity of the Arabidopsis root cells (Farmer et al., 2021). Such approaches should be expanded to other modalities, potentially gained at the same time from the same cell, to maximize dataset integration and to highlight the relationships existing between structural and chemical changes on the genomic DNA, somatic mutations, their impact on controlling gene expression and protein abundance. Considering the recent emergence of real multi-omics technology (e.g., analysis of gene expression and profile of chromatin accessibility from the same cell/nucleus using 10x Genomics technology), such technology needs to be expanded to other biological modalities to reveal the diverse and dynamic use of genomic information, proteome and metabolome of thousands of individual plant cells.

## Characterize Molecular Modalities at the Single-Cell Level in the Context of Plant Cell-to-Cell Interactions

Proximal interactions and distal communication between cells and organs play critical roles in plant biology. Local communication between two cells depends on the formation of plasmodesmata that connect the cytoplasm of neighboring cells to allow the exchange of proteins, metabolites, and nucleotidic sequences. For instance, the transportation of auxin and cytokinin between root cells via plasmodesmata plays a major role in controlling plant organ differentiation, such as the initiation of the formation of lateral root (Mellor et al., 2020) and legume nodule, plant organ resulting from the symbiotic interaction between legumes and Rhizobia (Fisher et al., 2018). Distal communication also plays a critical role in regulating biological processes. For instance, legume nodulation is controlled by the autoregulation of nodulation mechanism, a distal communication system between the canopy and the root of legume plants that regulates the formation of nodules and, as a consequence, nitrogen fixation efficiency (Kassaw et al., 2015; Wang et al., 2018; Suzaki and Nishida, 2019; de Bruijn, 2020).

Besides their proximal and distal interactions with other somatic cells, plant cells are also subject to interactions with a diverse population of microbes. Therefore, plant cells must constantly adapt their response upon recognition of symbiotic and pathogenic microbes notably by regulating cell-to-cell trafficking (Aung et al., 2020). Decades of work on plant microbes interactions revealed that the response of plant cells to microbial infection is complex and sequential. Upon recognition of the pathogen, a first response, the MAMP-triggered immunity response, is initiated. Later, the effector-triggered immunity response will allow the infected plant cell to initiate a more specific response to the microbe (Jones and Dangl, 2006; Naveed et al., 2020). These two types of immunity form the plant cell-autonomous immunity. Increasing the complexity of these interactions, the neighboring plant cells to an event of infection will trigger a non-cell-autonomous immunity response to minimize new events of infection by the same microbe. This immunity is activated upon communication between plant cells (Yan et al., 2019; Aung et al., 2020; Li et al., 2020; Zeng et al., 2020). Considering that different events of interaction and infection co-occur in a complex organ, the molecular characterization of the cell-autonomous and non-cell-autonomous responses of plant cells to a pathogenic infection remains challenging when conducted at the level of complex tissues and organs. Similar challenges are also faced by plant scientists when considering other complex cellular interactions including mutualistic symbiotic interactions between plants and microorganisms (e.g., legume nodulation and arbuscular mycorrhization) or when considering the interactions of plants with multicellular organisms, such as nematodes and insects. Single-cell approaches represent an attractive solution to reveal the cell-autonomous and non-cell-autonomous regulatory programs activated and repressed by the plant in response to microbial infection and pathogenic organisms. Ultimately, considering that the transcriptome of plant cells at different stages of infection will be captured and considering the development of performant computational tools to create transcriptomic trajectories (Qiu et al., 2017; Hao et al., 2020), the use of single-cell omics technology will clarify the sequential transcriptomic response of the plant cell to pathogenic infections.

To enhance our understanding of the biology of the plants as a complex and organized cellular system, the molecular attributes of each cell should be characterized in the context of the morphology of the tissue/organ. However, as mentioned above, a pre-requirement to the use of single-cell omics technologies is the dissociation of the tissue to access independent biological entities (i.e., cells or nuclei) and, consequently, the loss of the spatial organization of the cells in the tissue. This limitation is partially recovered by the use of performant dimensionality reduction methods (e.g., UMAP and t-SNE) that allow the annotation of the plant cells based on their molecular attribute. Therefore, the dividing/differentiating cells have been reported to be located in the center of dimensionality reduction maps while differentiated cells are located at their periphery (Ryu et al., 2019; Farmer et al., 2021).

## The Arabidopsis Root Single-Cell Transcriptome Highlights the Role of Cell-to-Cell Interactions in Controlling Root Hair Differentiation

**Figure 2:**
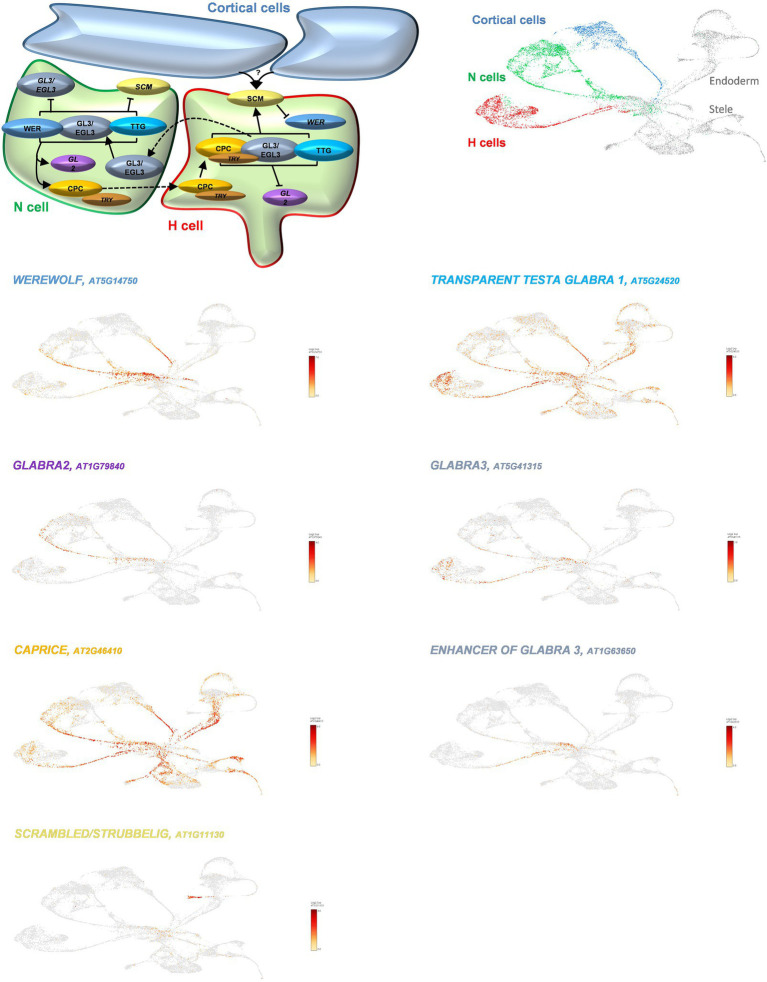
Transcriptional activity of the Arabidopsis genes playing a major role during root epidermal cell differentiation, a biological process that depends on intercellular communication (top left panel). The relative expression levels of the genes controlling Arabidopsis epidermal cell differentiation are highlighted in yellow/red color. The cortical, N, and H cell clusters are, respectively, highlighted in blue, green, and red on the top right panel. Shortly, a biological signal produced in high quantities by two cortical cells underlying a single H root epidermal cell is detected by the epidermal cell-localized leucine-rich repeat receptor SCM. This recognition notably leads to the repression of the expression of the *WER* gene and the initiation of epidermal cell differentiation. Based on the previous studies, *WER* and *GL2* are expected to be specifically expressed in the N cells (green cluster). Besides, we found that *WER* is also expressed in cortical cells (blue cells). As expected, the expression of *GL3* and *EGL3* is almost exclusively restricted to the H cells (red cells). Sc/sNucRNA-seq datasets also confirmed the transcriptional activity of *TTG1* in both N and H cells (green and red cells). However, *CPC* seems to have a ubiquitous transcriptional activity.

To evaluate the usefulness of single-cell transcriptomes in the context of cell-to-cell communication, we looked at the transcriptional activity of the Arabidopsis genes involved in the differentiation process and patterning of the root epidermal cells, a biological process that depends on intercellular communication between cortical and epidermal cells. The genes controlling the differentiation of the epidermal cells into H and N cells (i.e., trichoblasts and atrichoblasts, respectively) have been well characterized through a series of functional genomic studies (Salazar-Henao et al., 2016). Upon detection of a signal generated in large quantities by the two cortical cells underlying an H cell, the root epidermal leucine-rich repeat receptor SCRAMBLED (SCM) repressed the expression of the MYB transcription factor WEREWOLF exclusively in the H cells (Kwak et al., 2005; Kwak and Schiefelbein, 2007; Wang et al., 2019). Mining the recently published Arabidopsis single-cell/nucleus RNA-seq UMAP projections (Farmer et al., 2021), we found *WER* mostly transcriptionally active only in a subpopulation of atrichoblasts, and in the lower branch of the cortical cells (Figure 2). As expected, *WER* was not significantly expressed in the H cells. In the N cells, WER interacts with GLABRA3 (GL3), ENHANCER OF GLABRA3 (EGL3), and TRANSPARENT TESTA GLABRA (TTG) to induce the expression of *CAPRICE* (*CPC*), *TRIPTYCHON (TRY),* and *GLABRA2* (*GL2*) (Koshino-Kimura et al., 2005). Mining the single-cell/nucleus transcriptome, we observed the co-expression of *TTG*, *CPC*, *TRY*, and *GL2* genes, and, at a lower level, the expression of *GL3* and *EGL3* in the atrichoblast cluster (Figure 2). Besides, *GL3*, *EGL3*, *TTG,* and *CPC* are also expressed in the trichoblast cluster. This observation is supported by the role of these genes in controlling the differentiation of H cells notably by repressing the expression of *GL2* (Kurata et al., 2005). Indeed, we did not detect any *GL2* transcripts, nor *TRY*, into the cells and nuclei composing the trichoblast cluster as supported by previously published works (Rerie et al., 1994; Di Cristina et al., 1996; Masucci et al., 1996). This transcriptomic analysis at the single-cell level supports functional genomic studies showing the co-expression of major regulatory genes controlling the differentiation process and patterning of the root epidermal cells. However, the isolations of protoplasts or nuclei before conducting single-cell omics analyses necessarily lead to the loss of the physical interactions between cells. Therefore, the molecular information collected on isolated cells cannot be analyzed in the context of the relative position of the cells in the organ and their interactions with their neighboring cells.

## Perspectives

To gain a systems view of plant cellular communication, there is a need to quantify molecular modalities of individual cells in the context of the morphology of the organ analyzed, or at least to bridge information gained from single-cell approaches with the relative position of the cells.

Spatially resolved transcriptomics technologies offer an opportunity to access the transcriptome of cells or groups of cells in the context of the morphology of the organ analyzed. While spatial methods are now routinely applied in animal science, the plant scientific community starts to discuss their use to better understand gene regulation (Giacomello and Lundeberg, 2018; Gurazada et al., 2021). Spatial transcriptomics technologies will also facilitate the characterization of cell-type-specific molecular markers. While marker genes are well characterized in model plant species, such as *Arabidopsis thaliana* (e.g., Denyer et al., 2019; Jean-Baptiste et al., 2019; Ryu et al., 2019; Shulse et al., 2019; Zhang et al., 2019; Farmer et al., 2021), non-model species suffer from limited access to the cell-type-specific marker genes needed to properly annotate the various cell types composing an organ based in their transcriptomic information. The use of spatial transcriptomics technology on plant organ cross-sections will allow the characterization of cell-type-specific marker genes in the morphological context of the organ analyzed. This knowledge can be used to enhance the functional annotation of plant single-cell clusters; especially from plant species or organs where the number of functionally validated single-cell marker genes is limited.

Two strategies have been used to enable spatial transcriptomics analyses. On the one hand, Slide-seq (Rodriques et al., 2019) and Visium technology from 10x Genomics^®^ are based on the use of nucleotide spatial *barcodes arrayed on a slide. On the other hand,* High-Definition Spatial Transcriptomics (Vickovic et al., 2019) and Spatial Molecular Imaging technology from Nanostring^®^ offer very high-level resolution transcriptomes of complex organs. Applied to plant samples, these technologies will enable the accurate analysis of the differential use of the genomic information between plant cells and the impact of cell-to-cell interactions in controlling biological processes. However, it is important to acknowledge that spatial omics technologies suffer from several limitations. First, the resolution of the information gained might requires the use of computational methods for deconvolution of the Visium pots (e.g., the 10x Genomics Visium Gene Expression spots are 55 μm in diameter leading to the analysis of the transcriptome of several plant cells per spots; Andersson et al., 2020; Elosua-Bayes et al., 2021). Second, spatial omics technologies are currently almost exclusively restricted to the analysis of the transcriptome. To overcome this limitation, Liu et al. (2020a) recently developed Deterministic Barcoding in Tissue (DBiT-seq) technology allowing the quantification of transcripts abundance and the detection of proteins of interest in the context of the morphology of tissue (Liu et al., 2020a). To date, this method has been applied on mouse embryos and will likely require substantial optimization before implementing its use on plant samples. Such an approach will need to be expanded to cover additional molecular modalities to gain a deeper understanding of plant cell biology and to reveal the impact of these modalities on cell biology, physiology, and morphology.

## Data Availability Statement

The datasets presented in this study can be found in online repositories. The names of the repository/repositories and accession number(s) can be found at: https://www.ncbi.nlm.nih.gov/geo/, GSE155304.

## Author Contributions

ST drafted the manuscript. ML edited the manuscript. All authors contributed to the article and approved the submitted version.

## Conflict of Interest

The authors declare that the research was conducted in the absence of any commercial or financial relationships that could be construed as a potential conflict of interest.

## Publisher’s Note

All claims expressed in this article are solely those of the authors and do not necessarily represent those of their affiliated organizations, or those of the publisher, the editors and the reviewers. Any product that may be evaluated in this article, or claim that may be made by its manufacturer, is not guaranteed or endorsed by the publisher.
